# The tuberculosis vaccine challenge

**DOI:** 10.2471/BLT.23.020523

**Published:** 2023-05-01

## Abstract

The development of effective tuberculosis vaccines requires significant advances in knowledge and methods and a massive increase in investment. Tatum Anderson reports.

Like many of her peers, Dr Tereza Kasaeva was concerned to see an increase in the number of people falling ill with tuberculosis (TB) in 2021 after decades of steady decline. 

“An estimated 10.6 million people fell ill with the disease in 2021, up from 10.1 million in 2020,” says the director of the World Health Organization’s (WHO) Global TB Programme, adding that close to half a million of those people developed drug resistant forms of the disease. 

Also disquieting was the number of people dying, tuberculosis deaths reaching an estimated 1.6 million in 2021, up from an estimated 1.5 million in 2020 and 1.4 million in 2019.

The deteriorating epidemiological picture is putting key targets in question, including the targets set under the WHO End TB Strategy to reduce the global tuberculosis incidence rate by 80% and the number of tuberculosis deaths by 90% worldwide by 2030 relative to the 2015 baseline.

“Globally, in 2021 the TB incidence rate was only 10% lower than in 2015, and the number of TB deaths was down only 5.9%,” says Katherine Floyd, head of WHO’s tuberculosis monitoring unit. “We are well off track.”

Caused by *Mycobacterium tuberculosis*, tuberculosis is spread through the air when people with the pulmonary form of the disease (the most common) cough, sneeze or spit.

An estimated 10 million people develop tuberculosis each year, while around a quarter of the global population is already infected and carries the bacterium. Between 5–10% of carriers risk developing active tuberculosis in their lifetime.

There is a broad consensus about what is driving the negative trends. “The COVID-19 pandemic has clearly had a huge impact in terms of prevention (including vaccination), diagnosis and treatment,” says Kasaeva. “This, coupled with ongoing crises driven by armed conflict and climate change, has hampered progress.”

But as Kasaeva points out, other headwinds have been blowing for some time, among them persisting widespread poverty and associated undernourishment, inadequate health care, a steady increase in drug resistance, and a century-old vaccine that is only partially effective.

First used medically in 1921, the Bacillus Calmette–Guérin (BCG) has presented variable levels of efficacy in clinical trials, but it is generally agreed that, while providing partial protection for infants and young children against the development of severe forms of the disease, it does not protect adolescents and adults, the groups responsible for most transmission.

“We have waited more than a century for a new effective TB vaccine.”

Frank Cobelens

For Frank Cobelens, until recently executive chair at the Amsterdam Institute for Global Health and Development, addressing the lack of an effective tuberculosis vaccine is imperative. “Reaching key tuberculosis targets is going to require the development of a vaccine that is effective both before and after exposure, and across all age groups,” he says.

Cobelens recently finished working on a research and development roadmap for new tuberculosis vaccines for the European & Developing Countries Clinical Trials Partnership, a collaboration between countries in Europe and sub-Saharan Africa, supported by the European Union. He came away from the exercise with several concerns.

The first was the lack of different kinds of vaccine in early trials. “Ideally, you want multiple candidates coming at the problem in different ways in the preclinical and early clinical stages, so if one approach fails you have some kind of back-up,” he says.

There are currently at least 16 vaccines in the tuberculosis vaccine pipeline, five of which are in the final phase (phase III) of human trials. They all focus on a relatively narrow set of targets on the mycobacterium (antigens known to be pathogenic), and employ just three biotechnology platforms – live-attenuated vaccines (a weakened form of the live bacterium), deactivated or dead, and adjuvanted protein vaccines (vaccines containing a protein antigen, boosted by another substance to elicit the required immune response).

The latter has attracted considerable attention, partly because of its relative novelty, and partly because it has yielded some of the more promising candidates. One is candidate M72/ASO1E, which demonstrated 50% protection against TB after three years follow-up in a phase II trial which completed in 2018 and involved 3575 participants in Kenya, South Africa and Zambia.

The vaccine is now set to enter a phase III trial in high-incidence settings across Africa, with an estimated 26 000 participants. The trial will include people never infected and people with the latent form of the disease, to see if they are protected from developing the active form. Because the trial will require three years for recruitment and five for follow-up, it is unlikely to provide results until the start of the 2030s.

This is another problem identified by Cobelens: slowness. “We have waited more than a century for a new effective TB vaccine, and unfortunately we are going to have to wait several more years,” he says.

And he is not alone in making this observation. Suvanand Sahu, deputy executive director of the Stop TB Partnership is similarly frustrated at the pace of the research and development pipeline, all the more so having witnessed the way in which coronavirus disease 2019 (COVID 19) vaccine development was accelerated.

“We saw during the COVID-19 pandemic how, if you have urgency, if you have political will, and if you have the funding, the science and research can be fast-tracked, and you can get vaccines within a few months,” he says.

Of course, new biotechnologies were also key to the rapid development of COVID 19 vaccines, notable among them the use of messenger RNA (mRNA) to generate the production of antigens in the body required to elicit an immune response. Many are hoping to see the technology applied to tuberculosis vaccine R&D, while acknowledging the challenges faced.

“There is cautious excitement in the global tuberculosis community about mRNA vaccines, but TB and COVID-19 are very different diseases in terms of rates of transmission and pathophysiology,” says Mel Spigelman, president and chief executive officer of the Global Alliance for TB Drug Development.

No such vaccines have yet to emerge, but all eyes are on BioNTech (the private biotech company which partnered with Pfizer to manufacture one of the first COVID-19 vaccines) which is working on a multi-antigen ribonucleic acid (RNA) vaccine (now in Phase I trials) and Moderna, another private biotech firm which, in April 2022, announced a collaboration with the International AIDS Vaccine Initiative to develop a tuberculosis vaccine.

“Investment in vaccine research has to be stepped up.”

Tereza Kasaeva

Cobelens also cautions against too much excitement regarding possible breakthroughs, underlining the gaps in our basic understanding of the way the mycobacterium interacts with the human body. He also draws attention to the lack of valid preclinical models (typically, non-human animal preparations that mimic conditions in the human body) needed for research.

“The absence of validated preclinical models means it’s impossible to predict how a vaccine is going to behave in terms of protection until it’s in later trials,” Cobelens explains, adding that the absence of validated immunological correlates of protection (measurable biomarkers such as antibodies that are indicative of immune response) that can be used to predict clinical efficacy in trials is also a problem.

Sahu is hoping that a United Nations high-level meeting (UNHLM) on the fight to end tuberculosis which is planned for September 2023 will address these different gaps. “The theme of the meeting is advancing science, finance and innovation,” he says. “I hope that any eventual declaration will include specific language on accelerating R&D for tuberculosis vaccines.”

But he is also keen to see a significant increase in tuberculosis R&D funding. One of the main purposes of the high-level meeting is to review progress made since the 2018 meeting, when the UN agreed a target of 2 billion United States dollars (US$) per year for all tuberculosis research, including new diagnostics, treatments, and vaccines. As Sahu points out, that target has not been met. “TB R&D funding generally in 2021 was US$ 1 billion, which is half the amount proposed by the UNHLM,” he says.

WHO is also pushing for increased investment in tuberculosis vaccine research as part of a broader resource mobilization effort, as set out in its recently published *DG Flagship Initiative to #ENDTB 2023-2027*. The initiative aims to accelerate progress towards tuberculosis targets over the period 2023-2027, and calls for the licensing of at least one new tuberculosis vaccine by 2025. 

In addition, WHO Director-General Tedros Adhanom Ghebreyesus recently announced plans to establish a TB Vaccine Accelerator Council to fast track the development of and access to novel TB vaccines.

An indication of just how big an impact such a vaccine could have was provided by the WHO-commissioned, full value assessment of new TB vaccines, which was published by the Organization in December 2022. The assessment found that even at efficacy levels around 75%, a new vaccine could avert up to 110 million new cases and 12.3 million tuberculosis deaths by 2050.

“Investment in vaccine research has to be stepped up,” says Kasaeva. “We spent decades neglecting TB vaccine research at our peril. Now it is time for the international community to rally behind efforts to make TB vaccines a reality,”

**Figure:**
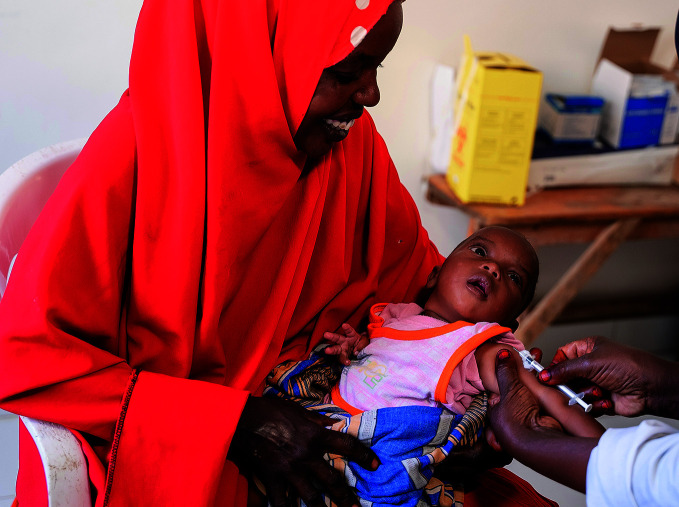
An infant is vaccinated with BCG at Gargaar Health Centre in Garowe, Somalia

UNICEF/Grace Ekpu

**Figure:**
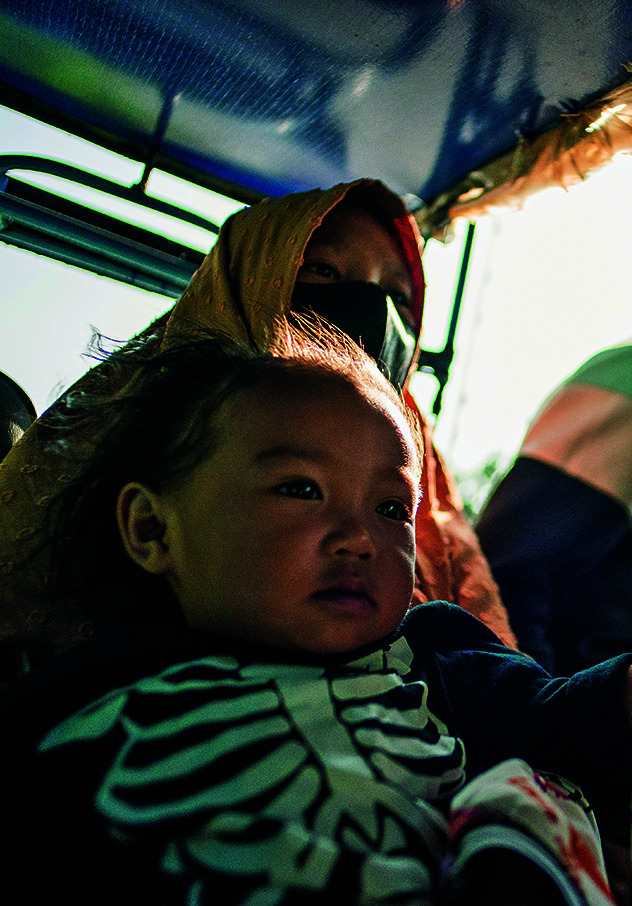
A “zero-dose” child (a child yet to receive any routine vaccine), travels with his mother to be vaccinated against tuberculosis and other diseases in Marantao, Philippines

UNICEF/Martin San Diego

